# Composition and conservation of the mRNA-degrading machinery in bacteria

**DOI:** 10.1186/1423-0127-18-23

**Published:** 2011-03-22

**Authors:** Vladimir R Kaberdin, Dharam Singh, Sue Lin-Chao

**Affiliations:** 1 Institute of Molecular Biology, Academia Sinica, Taipei 11529, Taiwan; 2 Department of Immunology, Microbiology and Parasitology, University of the Basque Country, UPV/EHU, Leioa, Spain; 3 IKERBASQUE, Basque Foundation for Science, 48011, Bilbao, Spain

## Abstract

RNA synthesis and decay counteract each other and therefore inversely regulate gene expression in pro- and eukaryotic cells by controlling the steady-state level of individual transcripts. Genetic and biochemical data together with recent in depth annotation of bacterial genomes indicate that many components of the bacterial RNA decay machinery are evolutionarily conserved and that their functional analogues exist in organisms belonging to all kingdoms of life. Here we briefly review biological functions of essential enzymes, their evolutionary conservation and multienzyme complexes that are involved in mRNA decay in *Escherichia coli *and discuss their conservation in evolutionarily distant bacteria.

## 1. mRNA turnover and its role in gene expression

**Figure 1:**
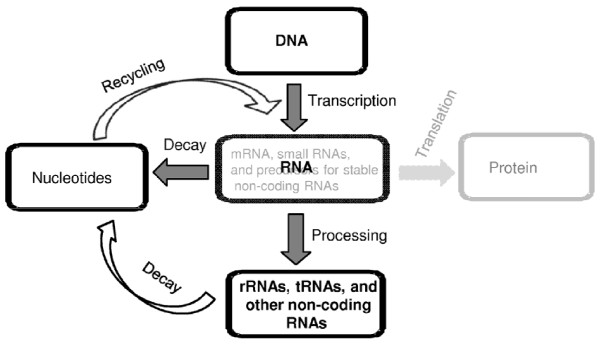
**RNA synthesis and turnover as part of the gene expression network in bacteria**. Different types of RNA (mRNAs, ribosomal and transfer RNA pre-cursors and various non-coding RNAs) either can directly be involved in translation (e.g. mRNAs) or undergo further processing (pre-cursors of stable RNA) or degradation (untranslated or poorly translated mRNAs) by the RNA decay machinery. The final products of RNA turnover, mononucleotides, are used for the next cycles of RNA synthesis (recycling).

In contrast to metabolically stable DNA serving as a storehouse of genetic information, the fraction of total RNA that delivers coding information to the protein-synthesizing machinery (i.e. mRNA) is intrinsically labile and continuously synthesized. The steady-state level of mRNA is tightly controlled enabling bacteria to selectively copy (transcribe) and decode genetic information pertinent to a particular physiological state (Figure 1). Since the steady-state level of mRNA can vary and is a function of RNA synthesis and decay, the control of mRNA stability plays an essential role in the regulation of gene expression. As transcription and translation are coupled in bacteria, the degree of their coupling can control the access of individual transcripts to the RNA decay machinery, thus influencing the rate of mRNA turnover. For more information about the crosstalk between translation and mRNA decay in bacteria and its regulation by environmental factors, we recommend some recent reviews (see [1-5]).

The ability of bacteria to rely on remarkably diverse metabolic pathways in order to adopt and strive in different environmental niches suggests that the nature and number of enzymatic activities involved in specific metabolic pathways including mRNA turnover can greatly vary from species to species. Hence, an analysis of the putative organization and composition of bacterial mRNA decay machineries that belong to phylogenetically distant species should enable us to gain critical insights into the evolution of RNA decay pathways and their conservation in bacteria. The main objective of this review was therefore to assess the evolutionary conservation of RNases and ancillary factors that are involved in mRNA turnover and briefly discuss their specific roles in this process.

## 2. Enzymes with major and ancillary functions in mRNA turnover and their phylogenetic conservation in bacteria

Early studies on RNA processing and decay in *E. coli*, a Gram-negative bacterium that belongs to the gamma division of proteobacteria, revealed several endoribonucleases (cleave RNA internally), exoribonucleases (sequentially remove mononucleotides from either the 5' or the 3'-end of RNA) and other RNA-modifying enzymes with important functions in mRNA turnover (Table 1). The specific roles of these enzymes as well as their functional homologues found in another model organism, the Gram-positive bacterium *Bacillus subtilis*, have been reviewed recently [5]. Here, we focus on the phylogenetic conservation of the major RNases (e.g., RNase E, polynucleotide phosphorylase, RNase II) and ancillary RNA-modifying enzymes (RNA pyrophosphohydrolase (RppH), poly(A) polymerase I (PAPI) and RNA helicase B (RhlB)) involved in the turnover of mRNAs in bacteria. Previous bioinformatic approaches have revealed that several mRNA-degrading enzymes are not strictly conserved and can be absent in some classes of bacteria [6,7]. The availability of new genomic data and discovery of novel RNases in bacteria prompted us to re-assess the phylogenetic conservation of these enzymes in bacterial species for which the sequence of the entire genome is available. The potential presence of mRNA degrading and mRNA-modifying ancillary enzymes was examined in all classes of bacteria by searching for the corresponding annotated genes and protein sequences available in the NCBI database http://www.ncbi.nlm.nih.gov/. The result of this analysis leads to several important conclusions regarding the nature and occurrence of RNases, ancillary enzymes (see 2.1 and 2.2) and their multienzyme assemblies (see 2.3) in evolutionarily distant species.

**Table 1:** Major ribonucleases acting on single-stranded (ss) or double-stranded (ds) regions of RNA and ancillary RNA-modifying enzymes (pyrophosphohydrolase, RppH; poly(A) polymerase I, PAPI; and DEAD-box RNA helicases) involved in RNA turnover in bacteria.

Endoribonucleases			
**Name**	**Essential for cell survival**	**Description of the reaction** **catalyzed**	**Specific functions *in vivo***

RNase E/G	Yes	Cleavage of A/U-rich ss regions of RNA yielding 5'-monophosphorylated products; 5'-end-dependent hydrolase	Ribosomal and transfer RNA processing, initiation of decay of non-coding and mRNAs, turnover of messenger, non-coding and stable RNA decay intermediates
RNase III	Yes	Endonucleolytic cleavage of ds regions of RNA yielding 5'-monophosphorylated products	Ribosomal and transfer RNA processing and mRNA processing and decay
RNases J1/J2*	RNaseJ1/Yes	Endonucleolytic cleavage of ss regions of RNA yielding 5'-monophosphorylated products; 5'-end-dependent hydrolase	RNA processing and decay in *B. subtilis*
RNase Y	Yes	Endonucleolytic cleavage of ss regions of RNA yielding 5'-monophosphorylated products; 5'-end-dependent hydrolase	Degradation of *B. subtilis *transcripts containing SAM-dependent riboswitches

**Exoribonucleases**			

**Name**	**Essential for cell survival**	**Description of the reaction** **catalyzed**	**Specific functions *in vivo***

RNase PH	No	tRNA nucleotidyltransferase	Exonucleolytic trimming of the 3'-termini of tRNA precursors
PNPase	No	(i) Phosphorolytic 3' to 5' exoribonuclease and (ii) 3'-terminal oligonucleotide polymerase activities	3' to 5' decay of ssRNA
RNase II	Yes	Exonucleolytic cleavage in the 3' to 5' direction to yield ribonucleoside 5'-monophosphates	Removal of 3'-terminal nucleotides in monomeric tRNA precursors, 3' to 5' exonucleolytic decay of unstructured RNAs
RNase R	No	Exonucleolytic cleavage in the 3' to 5' direction to yield ribonucleoside 5'-monophosphates	3' to 5' exonucleolytic decay of structured RNAs (e.g. mRNA and rRNA)
RNase J1/J2*	Yes	Exonucleolytic cleavage in the 5' to 3' direction to yield nucleoside 5'-monophosphates	5' to 3' exonucleolytic decay of *B. subtilis *RNAs
Oligoribo-nuclease	yes	Exonucleolytic cleavage of short oligonucleotides to yield nucleoside 5'-phosphates	Completion of the last steps of RNA decay

**Ancillary RNA-modifying enzymes**			

**Name**	**Essential for cell survival**	**Description of the reaction** **catalyzed**	**Specific functions *in vivo***

RppH	No	Removal of pyrophosphate groups from the 5'-end of triphosphorylated RNAs	Facilitation of endoribonucleolytic cleavages of primary transcripts by RNase E/G
PAPI	No	Addition of adenosines to the 3'-end of RNA	Facilitation of 3' to 5' exonuclolytic decay of structured RNAs by adding 3' poly(A) tails
DEAD-box helicases	No	ATP-dependent unwinding of ds regions of RNAs	Facilitation of the PNPase- dependent decay of structured RNAs

The presented classification of the enzymes and their functions *in vivo *were adopted from several enzyme databases (KEGG, http://www.genome.jp; EXPASY, http://us.expasy.org/enzyme/; and IntEnz, http://www.ebi.ac.uk/intenz/.*RNases J1/J2 possess both exo- and endoribonucleolytic activities.

### 2.1 Conservation and diversity of major enzymes controlling the endoribonucleolytic decay of mRNA

**Figure 2:**
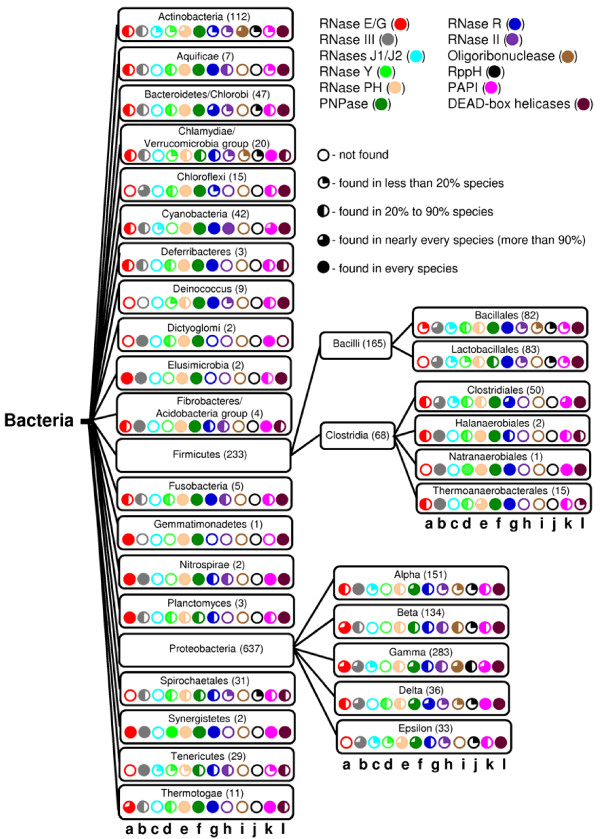
**The phylogenetic distribution of main ribonucleases (RNase E/G, RNase III, RNases J1/J2, RNase Y, RNase PH, PNPase, RNase R, RNase II, Oligoribonuclease) and ancillary RNA modifying enzymes (RppH, PAPI, DEAD-box helicases) involved in the disintegration and turnover of bacterial transcripts are indicated by colored filled circles (from 'a' to 'l', respectively)**. The percentage of organisms in each phylum/class of bacteria for which the presence of each particular enzyme has been predicted by searching the NCBI database is indicated by differentially colored circles. The data are compiled based on analysis of completely sequenced genomes (1217 complete genome sequences available by 4 November 2010). Draft assemblies of genomes and hypothetical proteins were excluded from the analysis.

Despite their indispensable functions in the processing of ribosomal and transfer RNA in *E. coli*, three major endoribonucleases, RNase E, RNase III and RNase P unequally contribute to mRNA decay. With few exceptions [8,9], the endoribonucleolytic decay of *E. coli *transcripts primarily involves RNase E and sometimes RNase III (reviewed in [10]). Moreover, previous studies of mRNA decay pathways in *E. coli *demonstrated the key role of RNase E, a member of the RNase E/G family of ribonucleases, in carrying out the first endoribonucleolytic cleavages initiating the ribonucleolytic decay of *E. coli *transcripts (reviewed in [11]). Although homologues of RNase E/G are predicted to be present in many bacterial species, they are either partially or completely absent in some phyla of bacteria (Figure 2). The lack of genes coding for this endoribonuclease suggests that either (i) the main functions of RNase E/G are occasionally taken over by other endoribonucleases or that (ii) RNase E/G is redundant for RNA processing and decay in some species.

The first possibility is supported by a recent analysis of RNA processing and decays in *B. subtilis *(class Firmicutes) [12-14]. Despite the discovery of RNase E-like cleavages in this bacterium [15], they were subsequently attributed to the action of two *B. subtilis *endoribonucleases (RNases J1 and J2) that bear primarily functional rather than sequence homology to their *E. coli *counterpart. Both RNase J1 and J2 were suggested to functionally represent RNase E/G in *B. subtilis *by mimicking the ability of RNase E to make endoribonucleolytic cuts in a 5'-end-dependent manner [12] as well as its property to form multienzyme complexes [13,14]. Interestingly, one recent study reported the existence and characterization of another *B. subtilis *endoribonuclease, RNase Y, and suggested that this enzyme is also functionally related to RNase E/G, in particular with regard to its role in mRNA turnover [16]. Consistent with this suggestion, we found that RNase Y appears to occur more frequently than RNases J1/J2 in the phyla that lack RNase E/G (Figure 2).

In contrast to Firmicutes, Actinomycetes and other phylas of bacteria whose members can apparently survive without RNase E/G by using its functional homologues, RNase Y and/or RNases J1/J2, some bacterial species seem to be able to carry out RNA processing and decay even in the absence of all these endoribonuclases (i.e., RNase E/G, RNase Y, and RNases J1/J2). Examples are some pathogenic bacteria that belong to the clades of Deinococcus, Dictyoglomy, Spirochaetales and Tenericutes. Many of these pathogens lack genes encoding not only the above endoribonucleases but also many exonucleases (see also 2.2).

Several studies revealed that the 5'-phosphorylation status of mRNA can control the efficiency of cleavages by RNase E/G homologues [17-21] as well as by RNases J1/J2 [12] and RNase Y [16]. As the *E. coli *pyrophosphohydrolase RppH (initially designated NudH/YgdP) is able to facilitate RNase E cleavage of primary transcripts by 5' pyrophosphate removal [22], we examined the presence of *nudH/ygdP *genes in genomes of phylogenetically distant bacteria. Despite the apparent absence of these genes in many classes of bacteria (Figure 2), their homologues that belong to the same family of Nudix hydrolases are known to be widely present in all three domains of life (reviewed in [23]). Therefore, it seems likely that the RNA pyrophosphohydrolase-mediated stimulation of mRNA decay in some bacterial species involves other members of the Nudix family of hydrolases.

### 2.2 Conservation and diversity of major enzymes controlling exoribonucleolytic decay of mRNA

A search for putative homologues of the three major mRNA-degrading exoribonucleases of *E. coli *(polynucleotide phosphorylase (PNPase), RNase II and RNase R) in other bacteria revealed that the corresponding genes can be found in nearly every class of bacteria (Figure 2). Although these observations suggest that mRNA decay in the majority of bacteria could be dependent on all three exoribonucleases, the actual contribution of each exoribonuclease to mRNA decay in these species may differ, as anticipated from previous studies of exonucleolytic decay of mRNA in *B. subtilis *(Firmicutes) and *E. coli *(Proteobacteria). These studies revealed that, in contrast to apparently similar roles of RNase II and PNPase in the degradation of *E. coli *mRNA [24], only PNPase plays a central role in the 3'-exonucleolytic decay of *B. subtilis *mRNA [25] with apparently less significant contribution of other exoribonucleases [25] including RNase PH [26], RNase R [27] and YhaM [28]. This is consistent with the previous finding that the 3'-to-5' exonuleolytic mRNA decay in *B. subtilis*, contrary to RNA turnover in *E. coli*, primarily proceeds through an "energy-saving" phosphorolytic pathway [29] mediated by PNPase. Further studies will be necessary to address systematically how phylogenetically distant bacteria combine different sets of exoribonucleases to carry out mRNA decay. Finally, given the high degree of phylogenetic conservation of PNPase and RNase II, it seems reasonable that one of the key ancillary enzymes, PAPI, which assists PNPase and RNase II in the degradation of structured RNAs, is likewise present in most of the bacteria, as shown in Figure 2.

### 2.3. Conservation of mRNA-degrading multienzyme complexes

**Figure 3:**
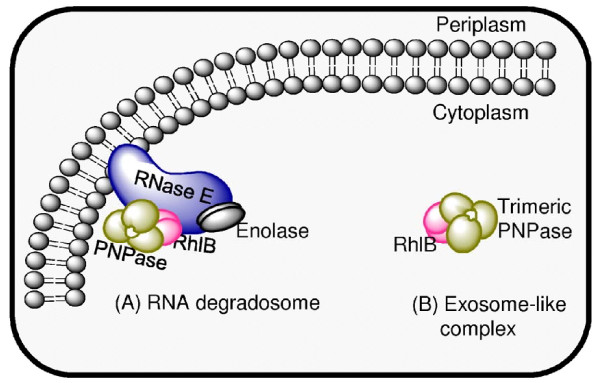
**Bacterial mRNA decay machineries**. (A) The RNA degradosome is a multicomponent ribonucleolytic complex that includes an endoribonuclease (RNase E), a 3'→5' exoribonuclease (polynucleotide phosphorylase (PNPase)), a DEAD-box RNA helicase (RhlB helicase), and the glycolytic enzyme enolase [31-33]). (B) In *E. coli*, PNPase is associated with the RhlB independently of the RNA degradosome to form an evolutionarily conserved RNA-degradation machine termed as the "bacterial exosome" [34,35]. This complex was shown to catalyze the 3'→ 5' exonucleolytic degradation of RNA using RhlB as cofactor to unwind structured RNA in an ATP-dependent manner.

Many *E. coli *mRNAs have relatively short half-lives (2-4 min) and are normally degraded *in vivo *without accumulation of intermediate products (reviewed in [30]), a phenomenon frequently referred to as the 'all-or-nothing' mechanism of mRNA turnover. The high processivity of mRNA decay is often discussed with reference to the coordinated action of ribonucleolytic enzymes and ancillary proteins that can associate with each other to form multienzyme ribonucleolytic complexes such as the *E. coli *degradosome (Figure 3A, [31-33]) and the bacterial exosome-like complex (Figure 3B) [34,35]. Analyses of the *E. coli *degradosome revealed that RNase E serves as a "scaffolding" protein, through the C-terminal part of which other interacting protein partners such as PNPase (exoribonuclease), RhlB (DEAD-box helicase) and enolase (glycolytic enzyme) are bound [36,37]. Consistent with these reports, the existence of functional interactions between the major components of the degradosome was confirmed *in vivo *[38-43] and *in vitro *[33,44]. Apart from binding to RNase E, two major components of the *E. coli *degradosome, PNPase and RhlB helicase, were shown to form a complex resembling the eukaryotic exosome, a multienzyme assembly with RNA-hydrolyzing and RNA-unwinding activities (reviewed in [35]). The formation and functions of this complex in *E. coli *may not be unusual as both enzymes appear to exist in excess to RNase E *in vivo *and therefore can be involved in alternative protein-protein interactions. However, the actual contribution of this complex to RNA metabolism in bacteria remains to be determined. mRNA molecules that are degraded by these multiprotein assemblies (i.e., degradosome and exosome) are simultaneously exposed to several ribonucleolytic and other RNA-modifying activities and therefore undergo fast and coordinated decay without accumulation of detectable amounts of intermediate products.

Although significant progress has been achieved in the characterization of the *E. coli *degradosome (reviewed in [45]), our current knowledge of the composition and properties of similar complexes in other bacteria is still very limited. A previous comparison of RNase E/G sequences revealed that the C-terminal half of *E. coli *RNase E (residues 499-1061), which is involved in protein-protein interactions with other major components of the *E. coli *degradosome, is poorly conserved among RNase E/G homologues [36]. Despite the overall lack of conservation, the PNPase-binding site of *E. coli *RNase E (residues 1021-1061, see [37]) is known to possess high similarity to a short amino acid sequence found in *H. influenza *Rd RNase E (residues 896-927, [36]). Moreover, this sequence is highly conserved among RNase E/G homologues of certain γ-proteobacteria (e.g., *Erwinia, Shigella*, and *Citrobacter*) and therefore is presently annotated in the NCBI database as the PNPase-binding domain. The conservation of this domain (although primarily in enterobacterial species) is also supported by a recent analysis of *Vibrio angustum *S14 RNase E [46]. This study defined the last 80 amino acids at the C-terminus of *Vibrio angustum *S14 RNase E as the potential site for PNPase binding and revealed the putative enolase-binding domain, a region also highly conserved amongst enterobacteria [47,48]. Collectively, the above findings and genomic data suggest that degradosome-like complexes are widespread in enterobacteria and organized in a similar manner.

In contrast to the apparently similar organization of enterobacterial degradosomes, their counterparts in other subclasses of γ-proteobacteria are less conserved. For instance, an analysis of the degradosome composition in the psychrotolerant γ-proteobacterium *Pseudoalteromonas haloplanktis *revealed that RNase E associates with PNPase and RhlB but not with enolase [49]. Moreover, a different degradosome-like complex consisting of RNase E, the hydrolytic exoribonuclease RNase R, and the DEAD-box helicase RhlE was purified from another psychrotrophic γ-proteobacterium, *Pseudomonas syringae *Lz4W [50]. As RNA structures are more stable at low temperatures and RNase R can degrade structural RNAs more efficiently than PNPase [51], the presence of RNase R (rather than PNPase) in this complex may be more advantageous for the degradosome-mediated decay in this psychrotrophic bacterium. RNase E-based degradosomes have also been isolated from other subclasses of proteobacteria. Hardwick and co-workers have recently isolated and characterized an RNase E-containing complex from the Gram-negative *α*-proteobacterium *Caulobacter crescentus *[52]. Apart from RNase E, this complex was found to contain PNPase, a DEAD-box RNA helicase and aconitase, an iron-dependent enzyme involved in the tricarboxylic acid cycle. One can envisage that, similar to its mycobacterial counterpart [53], *C. crescentus *aconitase may possess RNA-binding properties, and therefore can potentially modulate the efficiency and/or specificity of the degradosome-mediated RNA decay. More significant differences in the composition of degradosomes can be found in other *α*-proteobacteria. It has been shown that RNase E of *Rhodobacter capsulatis *forms a degradosome-like complex with two DEAD-box RNA helicases of 74 and 65 kDa and the transcription termination factor Rho [54]. Thus, the degradosome-dependent mRNA decay appears to involve different combinations of enzymatic activities even within the same class of bacteria.

In addition to analyzing the composition of degradosome complexes in Proteobacteria, some efforts were dedicated to identify degradosome-like complexes in Actinobacteria. These studies revealed that, similar to their *E. coli *counterpart, RNase E/G homologues can interact with PNPase in *Streptomyces *[55] and are able to co-purify with GroEL and metabolic enzymes in *Mycobacteria *[56]. The specific role of these polypeptides in RNA metabolism and the degree, to which their interaction with RNase E/G is conserved in Actinobacteria, remains to be established.

Aside from degradosome complexes that are believed to function in Proteobacteria and Actinobacteria, the existence of RNase E-based degradosomes in other classes of bacteria remains questionable. The small size (ca. 450-600 a.a., see Table 2) of RNase E/G homologues in many other classes of bacteria indicate that they primarily contain the evolutionarily conserved catalytic core of the enzyme and appear to lack regions serving as scaffolds for degradosome assembly [36,57].

**Table 2:** Bacterial RNase E/G homologues represented in the NCBI protein database

Phylum/Class	Length (aa)	Potential to form degradosome- like complex		Organisms tested for the presence of degradosome-like complexes/Reference
			
		Predicted based on the size of the protein	Experimentally verified	
Actinobacteria	463-1373	+	+	*S. coelicolor */[55] *M. tuberculosis; M. bovis */[56]
Aquificae	466-470	-	-	
Bacteroidetes/Chlorobi	503-570	-	-	
Chlamydiae/Verrucomicrobia group	510-554	-	-	
Cyanobacteria	602-808	-	-	
Deferribacteres	507	-	-	
Elusimicrobia	488	-	-	
Fibrobacteres/Acidobacteria group	511	-	-	
Firmicutes				
Bacilli	441-615	-	-	
Clostridia	393-571	-	-	
Fusobacteria	432-458	-	-	
Gemmatimonadetes	520	-	-	
Nitrospirae	514-522	-	-	
Planctomycetes	509-588	-	-	
Proteobacteria				
Alpha	411-1123	+	+	*R. capsulatus*/[54] *C. crescentus *[52]
Beta	394-1125	+	-	
Gamma	410-1302	+	+	*E. coli*/[32,33] *P. syringe*/[50] *V. angustum *S14 RNase E [46] *P. haloplanktis *[49]
Delta	486-926	+	-	
Synergistetes	495-547	-	-	
Thermotogae	454-481	-	-	

Interestingly, recent studies demonstrated that the Gram-positive bacterium *B. subtilis *(Firmicutes) possesses degradosome-like complexes, in which RNase E is represented by its functional homologues, RNases J1/J2 and RNase Y, interacting with PNPase, phosphofructokinase and enolase [13]. Further characterization of these complexes and elucidation of their specific roles in mRNA decay in *B. subtilis *and related species can offer many important insights into the mechanisms underlying mRNA decay in Firmicutes, the largest group of Gram-positive bacteria that have been studied so far [58].

## 3. Current unified model for mRNA decay pathways in *E. coli*

### 3.1 Both endo- and exoribonucleases act cooperatively to control mRNA decay

**Figure 4:**
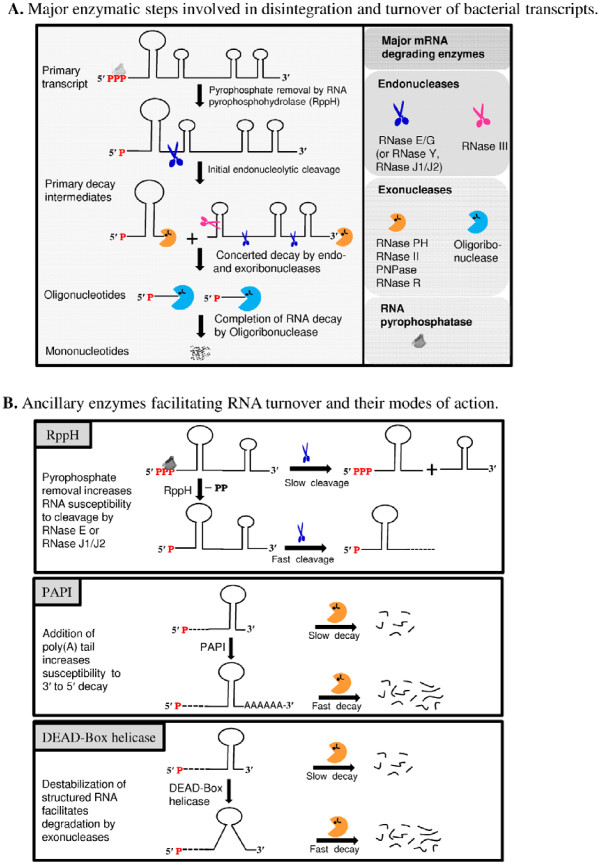
**Current unified model of mRNA decay pathways in *Escherichia coli***. (A) Schematic representation of major enzymatic steps involved in the disintegration and complete turnover of primary transcripts in *E. coli*. The decay of a regular transcript is usually initiated by endonucleolytic cleavage to generate primary decay intermediates that are further converted to short oligoribonucleotides by the combined action of exo- and endoribonucleases. The oligoribonucleotides are further degraded into mononucleotides by oligoribonuclease. (B) Ancillary enzymes facilitating mRNA turnover and their modes of action. Degradation of mRNA can be stimulated via pyrophosphate removal by RppH, which converts 5'-triphosporylated primary transcripts into their monophosphorylated variants, thus facilitating their endoribonucleolytic cleavage by RNase E [22,76] or by RNases J1/J2 [12] or by RNase Y [16] in *B. subtilis*. As the action of exoribonucleases can be inhibited by 3'-terminal stem-loop structures, two groups of ancillary RNA-modifying enzymes, PAPI and RhlB, help exonucleases to overcome this inhibitory effect. PAPI exerts its action by adding short stretches of adenosine residues, thereby facilitating exonuclease binding and subsequent cleavage of structured RNAs [10]. Enzymes of the second group, DEAD-box helicases such as *E. coli *RhlB, increase the efficiency of the exonuclease-dependent decay by unwinding double-stranded RNA regions in an ATP-dependent fashion.

Despite phylogenetic conservation (Figure 2) and their apparent diversity (for a review, see [10]), mRNA decay pathways in *E. coli *are believed to include a number of common enzymatic steps catalyzed by ribonucleases and several ancillary mRNA-modifying enzymes. To discuss the role of each enzyme, we will refer to a unified model of mRNA turnover. According to this model (Figure 4A), conversion of *E. coli *mRNAs into their primary decay intermediates is frequently initiated by endoribonucleolytic cuts catalyzed by endoribonucleases specific for single- (e.g., RNase E/G) or double-stranded (RNase III) RNA. This step can be preceded (but not always, see [59]) by pyrophosphate removal (see below). During the initial endoribonucleolytic step, bacterial RNase E/G (or its functional homologues, RNases J1/J2 or RNase Y) attacks the full-length monophosphorylated (or sometimes triphosphorylated [59]) mRNAs to generate primary decay intermediates that are further degraded cooperatively by the combined action of endo- and exoribonucleases (Figure 4A). In *E coli*, the later steps of mRNA decay were shown to involve PNPase and RNase II, or occasionally RNase R [51,60], which further degrade mRNA decay intermediates to yield short oligonucleotides that are, in turn, converted to mononucleotides by oligoribonuclease [61].

### 3.2 Ancillary enzymes facilitate mRNA turnover by assisting ribonucleases

In addition to the major degrading enzymes, a number of ancillary mRNA-modifying enzymes can facilitate mRNA turnover (Table 1). In fact, pyrophosphate removal at the 5'-end and addition of a single-stranded, poly(A) extension at the 3'-end are two critical steps in the mRNA decay pathway promoting mRNA cleavage in *E. coli *and presumably in other proteobacteria. In general, however, the participation of these enzymes in mRNA decay in some bacterial species or organelles is not required (see section **2**). One of these enzymes, RppH, was shown to accelerate mRNA decay by converting the 5'-triphosphate group of primary transcripts to 5' monophosphate, thereby rendering mRNA species that are more efficiently recognized and cleaved by RNase E [17,18] and RNase G [19].

Unlike RppH, whose action promotes endoribonucleolytic cleavages, some mRNA-modifying enzymes can stimulate degradation by 3' to 5' exonucleases (reviewed in [62,63]). Previous work has shown that the 3' to 5' degradation of transcripts by PNPase and RNase II in *E. coli *proceeds only efficiently on unstructured mRNAs and is impeded by stable stem-loop structures occurring internally (e.g., in intergenic regions of polycistronic transcripts such as REP stabilizers found in the *malEFG *and many other intergenic regions [39]) or at the 3' end of bacterial transcripts (i.e., transcription terminators [64]). These structures typically cause exoribonuclease stalling and subsequent dissociation of exoribonucleases from decay intermediates (reviewed in [62,63]). To prevent accumulation of decay intermediates that are resistant to 3' to 5' degradation by exoribonucleses, *E. coli *and apparently other bacteria employ a mechanism that increases the susceptibility of an mRNA decay intermediate to exonucleases by adding a poly(A) tail to its 3' end (Figure 4B). Consequently, repetitive cycles of poly(A) addition carried out by PAPI combined with exonuclease-catalyzed trimming was shown to result in the complete digestion of structured RNAs by either PNPase or RNase II *in vitro *[65]. Consistent with these findings, mRNA decay in a mutant lacking functional PAPI results in the accumulation of intermediate products of mRNA decay [64,66-69], thus indicating that the addition of poly(A) tails is indeed required for the normal mRNA turnover *in E. coli*. Because several aspects of poly(A)-assisted mRNA turnover including its role in the decay of stable RNA fall beyond the scope of this review, the interested reader is referred to other work covering this topic [70].

In *E. coli*, the exonucleolytic decay of highly structured RNAs can also be assisted by the RhlB (Figure 4B). This enzyme unwinds RNA structures in an ATP-dependent manner and therefore facilitates their degradation by exonucleases *in vivo *[39] and *in vitro *[33,34]. Moreover, RhlB is an integral part of the multienzyme RNA degradosome and exosome-like complexes and believed to exert its functions primarily as component of the mRNA decay machinery.

## 4. Conclusion and perspectives

A previous analysis of RNA processing/decay pathways in several distantly-related bacterial species including the two major model organisms, *E. coli *(Proteobacteria) and *B. subtilis *(Firmicutes) has identified the key ribonucleases involved in mRNA turnover in bacteria (reviewed in [5]). Herein, a search for their homologues in bacteria with completely sequenced genomes revealed that many components of the bacterial mRNA decay machinery (RNase III and three major exoribonucleases, PNPase, RNase II and RNase R) as well as PAPI and RhlB) are highly conserved across the bacterial kingdom (see Figure 2). In contrast, the major endoribonucleases RNase E/G, RNases J1/J2, and RNase Y possess only functional (but not sequence) conservation. Although they were found only in particular classes of bacteria, at least one of them is present in nearly every species. Thus, although RNA processing/decay in phylogenetically distant bacterial species is not necessarily carried out by the same set of ribonucleolytic enzymes (see previous sections), the minimal set of enzymatic activities (at least one functional homologue of RNase E/G and one 3' to 5' exoribonuclease) required for mRNA turnover in prokaryotic organisms is likely conserved in a vast majority of bacterial species.

Surprisingly, the number of enzymes with potential roles in RNA processing and decay is dramatically reduced in several intracellular pathogens possessing relatively small (less than 1 Mbp) genomes (e.g., *Mycoplasma *(Tenericutes), *Rickettsia *(α-Proteobacteria) and *Chlamydia *(Chlamydiae/Verrucomicrobia group)). In contrast to the presence of seven distinct exoribonucleases in *E. coli*, only one of them can be found in Mycoplasma (subclass *Mollicutes *(*Tenericutes*)). Analysis of RNA metabolism in *Mycoplasma genitalium *suggests that exonucleolytic decay in this bacterium can be accomplished by a single exoribonuclease, RNase R [71]. Another prominent feature of Mycoplasma is the lack of genomic sequences potentially encoding a homologues of *E. coli *PAPI known to catalyze the addition of poly(A) to the 3' end of *E. coli *transcripts [72]. The lack of this enzyme is consistent with the recent finding that demonstrated the absence of polyadenylated RNA in Mycoplasma [73]. Although the poly(A)-dependent enhancement of mRNA decay is likely redundant for some intracellular pathogens, it seems to be more important in some Proteobacteria and Firmicutes, as it can offer an additional mean to control the efficiency of mRNA turnover. In other words, unlike pathogens that continuously reside in host cells, bacteria that strive in highly diverse and continuously changing environments (e.g., *Escherichia coli*) use a large number of ribonucleases and ancillary mRNA-modifying enzymes such as poly(A) polymerases to efficiently regulate mRNA stability in response to environmental signals. Future studies addressing the main differences between the mechanisms of mRNA decay of intracellular pathogens and the currently used model organisms (*E. coli *and *B. subtilis*) may lead to important insights concerning the evolution of the mRNA decay machinery in bacteria.

Similar to other essential cellular processes controlling inheritance and expression of genetic information (i.e., DNA replication, transcription and translation), mRNA decay was found to be carried out by multienzyme complexes, several of which have been isolated from Proteobacteria, Actinobacteria and Firmicutes over the last two decades. The existence of functional interactions between the major components of the *E. coli *degradosome and their impact on mRNA turnover [38-43] suggest that multienzyme complexes (instead of a pull of non-interacting enzymes) are favorable for attaining a higher efficiency of mRNA decay. The role of similar complexes in other bacteria is still poorly defined. Moreover, we do not know to which degree RNase E/G-based degradosomes resemble their counterparts containing RNases J1/J2 or RNase Y existing in many other classes of bacteria. Likewise, the mechanisms modulating the composition, activity and specificity of these multienzyme assemblies in response to changing physiological conditions remain largely unknown and merit further analysis. Finally, although the last step of mRNA decay in *E. coli *has been shown to be accomplished by oligoribonuclease encoded by the *orn *gene [61], this gene is apparently absent in many other bacterial species (Figure 2). A search for activities that can degrade RNA oligoribonucleotides in Firmicutes lacking sequence homologues of *E. coli *oligoribonuclease led to the discovery of *B. subtilis *Ytq1 [74]. This enzyme possesses an oligoribonuclease-like activity and is able to complement the *E. coli orn *mutant; homologues of its gene are present in many bacteria [75]. Although Ytq1 can be considered as a functional homologue of oligoribonuclease, further efforts are needed to disclose the nature and distribution of functional homologues that may exist in bacterial species lacking both oligoribonuclease and Ytq1.

## Competing interests

The authors declare that they have no competing interests.

## Authors' contributions

The manuscript was prepared by VRK, DS and SL-C. All authors read and approved the final manuscript.
